# All-Trans Retinoic Acid Impacts Early Palatal Shelves Development via the Wnt and TGF-β Signaling Pathways

**DOI:** 10.3390/biomedicines13112836

**Published:** 2025-11-20

**Authors:** Yaping Ma, Binqing Wang, Shikang Gao, Tao Song

**Affiliations:** 1 Center for Cleft Lip and Palate Treatment, Plastic Surgery Hospital, Chinese Academy of Medical Sciences and Peking Union Medical College, Beijing 100144, China; m17853138339@163.com (Y.M.); gsk8130@163.com (S.G.); 2 Department of Plastic Surgery, Haidian District Maternal and Child Health Care Hospital, Beijing 100093, China; wangbinqing@126.com

**Keywords:** atRA, cleft palate, Wnt, TGF-β, proliferation, migration

## Abstract

**Background/Objectives:** All-trans retinoic acid (atRA), a potent derivative of vitamin A, is recognized as a significant teratogen for inducing cleft palate in both humans and mice. The molecular mechanisms underlying it remain intricate and incompletely elucidated. The advent of single-cell sequencing technology offers novel methodologies to investigate the mechanisms by which atRA induces cleft palate. **Methods:** In this study, we use C57BL/6 mice to conduct cleft palate models, comprising a control group and an atRA-exposed group. Palatal shelves were collected at embryonic day 12.5 (E12.5) for 10x single-cell sequencing analysis to discern and compare the cellular and molecular disparities between the two groups. Validation of the findings was performed using Quantitative real-time polymerase chain reaction and Western blot techniques. **Results:** The findings indicate that at E12.5, atRA predominantly affects the mesenchymal and epithelial cells of the palatal shelves, inhibiting cellular proliferation and migration. The primary mechanism of atRA’s effect involves modulation of the Wnt and TGF-β signaling pathways. Furthermore, the Ppp1r14b gene was identified as a critical mediator in atRA’s interaction with these pathways. **Conclusions:** This study provides a more comprehensive understanding of the mechanisms underlying atRA-induced cleft palate formation. It highlights the significance of the Wnt and TGF-β pathways, as well as the Ppp1r14b gene during this procedure.

## 1. Introduction

Vitamin A is one of the most important trace elements for maintaining human physiological activities. The main active components in the human body include all-trans retinoic acid (atRA) and 11-cis-retinal. Notably, 11-cis-retinal plays an essential role in visual function, while atRA is involved in the regulation of gene transcription [1,2]. However, excessive exposure to atRA during human pregnancy can cause abnormal embryonic development, with teratogenic effects primarily manifesting as craniofacial deformities, such as cleft palate, impaired neural tube closure, and cardiac defects [3,4]. Among craniofacial deformities, cleft palate has been widely studied due to its complex clinical sequelae. Epidemiological data show that 20–30% of children with cleft palate have persistent palatopharyngeal insufficiency following surgical repair [5], leading to articulation disorders and swallowing dysfunction, accompanied by secondary complications such as otitis media, hearing loss, and malocclusion [6,7]. In addition to physiological abnormalities, cleft palate patients are also exposed to a higher risk of psychological conditions, such as anxiety and depression, compared to ordinary people. This imposes a significant burden on their families and society [8]. Therefore, elucidating the molecular mechanisms underlying the formation of cleft palate has significant implications for global public health. Due to the similarity in secondary palate development between mice and humans, mice are commonly used as model animals for studying secondary palate development; specifically, atRA-induced cleft palate mouse models are widely utilized in this field [9]. The critical window period for mouse palate development is embryonic days 12.5–16.5 (E12.5–E16.5). E12.5 marks the onset of secondary palate development. The present study employed a single-cell sequencing platform to perform high-precision single-cell transcriptome sequencing (10x Genomics Chromium system) on secondary palatal tissues from E12.5 mouse embryos, comparing the normal group to the atRA-exposed group. This approach aims to provide deeper insights into the cellular and molecular alterations associated with cleft palate development, offering a foundation for further research into its pathogenesis.

## 2. Materials and Methods

### 2.1. Animals and All-Trans Retinoic Acid Exposure

The animal experiment protocol and procedures in this study were approved by the Laboratory Animal Ethical Committee of Plastic Surgery Hospital in Beijing, China. The method of this study in building animal models has been outlined in our previous research [10]. Pregnant C57BL/6J female mice were euthanized by cervical dislocation at E12.5. After dissecting the embryonic mice, the palatal shelves were isolated under a 10x microscope.

### 2.2. Tissue Dissociation and Single-Cell Sequencing

The palatal shelves were collected from both groups at E12.5, and a pooled single-cell suspension was processed using the 10x Chromium system to create single-cell gel beads in emulsion. The 10x Genomics Chromium single-cell 3′ v3.1 kit was utilized for library preparation. Sequencing was conducted on the NovaSeq 6000, PE150 X10 instrument (Illumina, San Diego, CA, USA).

### 2.3. Data Quality Control and Clustering

Quality control of the single-cell transcriptomic data and reads processing were performed using the Cell Ranger pipeline (v3.1.0). A gene expression matrix was generated using a unique molecular identifier (UMI). Then, the Seurat package (v5.3.0) [11] was used for further quality control. Harmony was utilized for data integration, performing standardization, normalization, and batch effects removal. Subsequently, the data were integrated using 2000 highly variable genes, and principal component analysis (PCA) was conducted. The top 30 principal components at the resolution of 1 were selected. Thereafter, the marker genes for each cluster were identified using the FindAllMarkers function.

### 2.4. Differentially Expressed Genes and Pathway Enrichment Analysis

Differentially expressed genes (DEGs) between the two groups were identified using the FindMarkers function in the Seurat package. DEGs were screened based on log_2_FC > 0.5 and *p* value < 0.01. Subsequently, DEGs were subjected to Gene Ontology (GO) analysis, including biological process (GOBP) and molecular function (GOMF), as well as Kyoto Encyclopedia of Genes and Genomes (KEGG) pathway enrichment analysis. These analyses were performed with R packages Apear (v1.0) [12] and clusterProfiler (v4.10.1) [13]. Pathways with a *p* value < 0.05 were considered statistically significant.

### 2.5. Estimation of Pathway Activity

Pathway activity in the two groups was estimated using the decouple R package (v 2.9.7) [14]. The results were visualized using a circular heatmap.

### 2.6. Analysis of Intercellular Communication

The mouse database in the CellChat package (v1.6.1) [15] was used to analyze the interactions among various cell types across different groups. This analysis incorporated the “Secreted Signaling,” “Cell–Cell Contact,” and “ECM-Receptor” modules. NicheNet package (v2.2.0) [16] was utilized to further analyze the predicted ligands, leading to expression changes in the specific cell types.

### 2.7. Cell Cycle Analysis

The R package tricycle (v1.10.0) [17] was utilized to analyze the cell cycle by scoring each cell based on the expression of G2/M, G2, S, G1/S, and M/G1 phase marker genes.

### 2.8. hdWGCNA Analysis

The R package High Dimensional Weighted Gene Co-expression Network Analysis (hdWGCNA) (v0.4.4) [18] was utilized to identify gene modules exhibiting high correlation with different groups within specific cell types. This analysis also enabled the identification of key genes and transcription factors within each module.

### 2.9. Simulation of Hub Gene Knockouts

To further explore the potential roles of hub genes in the pathogenesis of specific cell types, simulated knockout analysis was performed using the scTenifoldKnk package (v1.0.2) [19]. This approach enabled the identification of the most significant genes among the gene modules.

### 2.10. Quantitative Real-Time Polymerase Chain Reaction (qRT-PCR)

RNA was extracted from tissue samples using Trizol reagent (Invitrogen, Carlsbad, CA, USA) following the manufacturer’s instructions. The RNA was reverse-transcribed utilizing the PrimeScript RT Reagent Kit (Takara, RR037A, Kusatsu, Japan). We conducted real-time quantitative PCR utilizing SYBR Green I Master (Roche, Basel, Switzerland)) in a reaction volume of 20 µL. To quantify mRNA levels, glyceraldehyde-3-phosphate dehydrogenase (Gapdh) was employed as an internal reference standard. Table S1 lists the primers for both target and reference genes, with gene quantification analyzed using the 2-DDCT method.

### 2.11. Western Blot

Protein extraction and Western blot analysis were performed in order to measure the expression of Ppp1r14b proteins. Cell lysates were prepared using RIPA lysate (Beyotime, Beijing, China), and the protein concentration of the samples was measured with the BCA Protein Assay Kit (Beyotime, Beijing, China). Samples (approximately 40 µg) were separated by 10% Sodium dodecyl sulfate polyacrylamide gel electrophoresis (SDS-PAGE) and transferred to Polyvinylidene Fluoride (PVDF) membranes. The membranes were blocked in 5% non-fat milk for 2 h before being incubated with primary antibodies at 4 °C overnight. The primary antibodies included anti-Ppp1r14b (1:500, Affinity Biosciences, 18476-1-AP, Cincinnati, OH, USA), anti-Gapdh (1:3000, Proteintech, 60004-1-1g, Chicago, IL, USA). The membranes were then rinsed with TBST and incubated with the HRP-conjugated secondary antibodies (1:1000, Beyotime, A0208, Beyotime, Shanghai, China) at room temperature for 2 h. The iBright imaging system (Tanon, Shanghai, China) was utilized to obtain images, and Image J software (v1.53f, NIH, Bethesda, MD, USA) was used to quantify the expression of proteins.

### 2.12. Statistical Analysis

Both the control and atRA-exposed groups consisted of three biological repetitions, each with three technical replicates to ensure accuracy and reproducibility. Statistical analysis was performed using R software, employing an unpaired *t*-test to compare the atRA-exposed and control groups. Data were presented as mean ± SD in triplicate (*n* = 3). Significance was indicated by “*”, “**” and “***” for *p* < 0.05, *p* < 0.01, and *p* < 0.001, respectively.

## 3. Results

### 3.1. All-Trans Retinoic Acid Significantly Impacts the Mesenchymal and Epithelial Cells in the Initial Phase of Mouse Palatal Shelves Development

**Figure 1:**
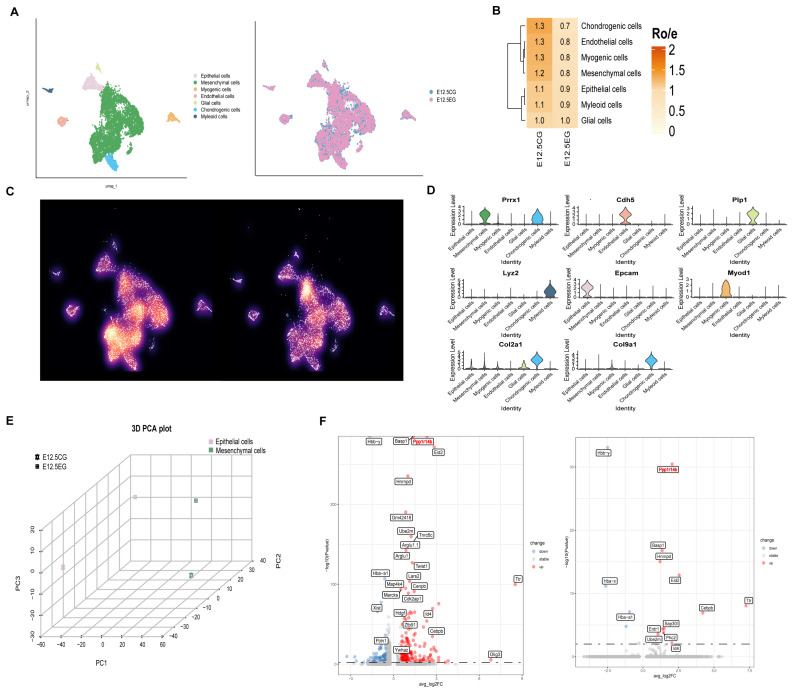
Single-cell altas of control group and atRA-exposed group. (**A**) UMAP visualization of major cell types by color (left) and cell type distribution by group (right). (**B**) Group preference of each cell type measured by R_O/E_. (**C**) Galaxy plots illustrate cell density within UMAP space, comparing control group (left) to atRA-exposed group (right). Darker colors represent lower density, whereas brighter colors indicate higher density. (**D**) Violin plots showing expression of marker genes for each cell type. (**E**) PCA. (**F**) Volcano plots of DEGs between different groups in mesenchymal cells (left) and epithelial cells (right), the dashed line represents the value of −log10(*p*-value) when *p*-value = 0.05.

To explore the impact of atRA at E12.5, single-cell RNA sequencing was performed on secondary palate samples from both the control and atRA-exposed groups, yielding 5920 and 7088 cells, respectively. Unsupervised analysis and specific marker gene expression identified several cell types: neural crest-derived mesenchymal cells (*Prrx1*), endothelial cells (*Cdh5*), glial cells (*Plp1*), myeloid cells (*Lyz2*), epithelial cells (*Epcam*), myogenic cells (*Myod1*), and chondrogenic cells (*Col2a1* and *Col9a1*), as displayed in (Figure 1A,D). Considering the pivotal roles of epithelial and mesenchymal cells in palatal shelves development [2], and given that these two cell types represent the predominant cellular populations (Figure S1A), we analyzed their distribution across the control and atRA-exposed groups. The analysis revealed a markedly lower enrichment of both mesenchymal and epithelial cells in the atRA-exposed group (Figure 1B,C). Therefore, subsequent investigations primarily focused on the alterations in these cell populations following atRA exposure. Three-dimensional PCA revealed significant differences in spatial distribution between the control and atRA-exposed groups, indicating significant changes in gene expression following atRA exposure (Figure 1E). Thereafter, differential gene expression analysis was conducted, identifying 283 DEGs in mesenchymal cells and 15 DEGs in epithelial cells. Compared to the control group, upregulated genes in the atRA-exposed group were enriched in serine kinase activity (*Map4k4*), and the Wnt (*Trim44*) and TGF-β (*Twist1*, *Cebpb*, and *Id4*) signaling pathways [20], among these, the protein phosphatase inhibitor *Ppp1r14b* exhibited the most significant differential expression in both cell types (Figure 1F). These findings indicate that atRA may impact palatal shelves development by affecting kinase and phosphatase activities and modulating the Wnt and TGF-β pathway.

### 3.2. All-Trans Retinoic Acid Exposure May Influence Mesenchymal and Epithelial Cells by Modulating Wnt and TGF-β Pathways

**Figure 2:**
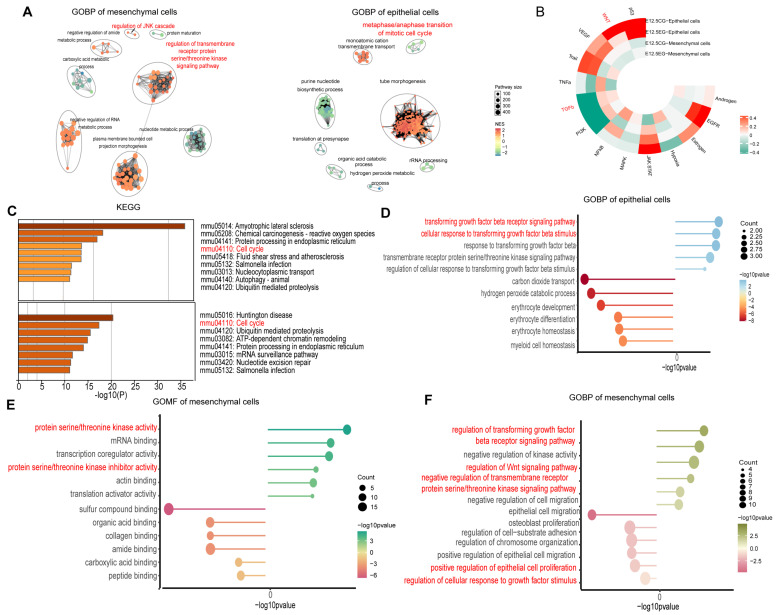
Functional enrichment analyses of DEGs in mesenchymal and epithelial cells. (**A**) GOBP enrichment analyses of DEGs in mesenchymal cells (left) and epithelial cells (right). (**B**) Comparison of pathway activity of mesenchymal cells and epithelial cells in two groups. (**C**) KEGG enrichment analyses of DEGs in mesenchymal cells (up) and epithelial cells (down). (**D**) GOBP enrichment analyses of upregulation and downregulation of DEGs in epithelial cells after exposure to atRA. (**E**) GOMF enrichment analyses of genes upregulated and downregulated among DEGs in mesenchymal cells after exposure to atRA. (**F**) GOBP enrichment analyses of genes upregulated and downregulated among DEGs in mesenchymal cells after exposure to atRA.

GOBP pathway enrichment analyses were conducted to identify key pathways in mesenchymal cells, showing enrichment in serine/threonine kinase signaling, JNK cascade regulation, and kinase activity. KEGG analyses further highlighted the cell cycle as an enriched pathway (Figure 2A,C and Figure S1B). In mesenchymal cells, upregulated genes in the atRA-exposed group were associated with inhibition of cell migration and TGF-β signaling, whereas downregulated genes were associated with epithelial cell proliferation and fibroblast growth (Figure 2E,F). Similar analyses in epithelial cells revealed enrichment in serine/threonine kinase signaling, phosphatase activity, TGF-β pathways, and cell cycle regulation (Figure 2A,C,D and Figure S1B,C). Consequently, atRA was hypothesized to regulate cell proliferation, cell cycle, and migration via the Wnt and TGF-β pathways. Subsequently, the “decoupleR” package was utilized to assess pathway activity, revealing that the Wnt pathway was significantly inhibited in both epithelial and mesenchymal cells of the atRA-exposed group. TGF-β is slightly inhibited in mesenchymal cells but shows little change in epithelial cells (Figure 2B), suggesting a complex regulatory mechanism.

### 3.3. All-Trans Retinoic Acid Inhibits the Proliferation and Migration of Mesenchymal and Epithelial Cells

**Figure 3:**
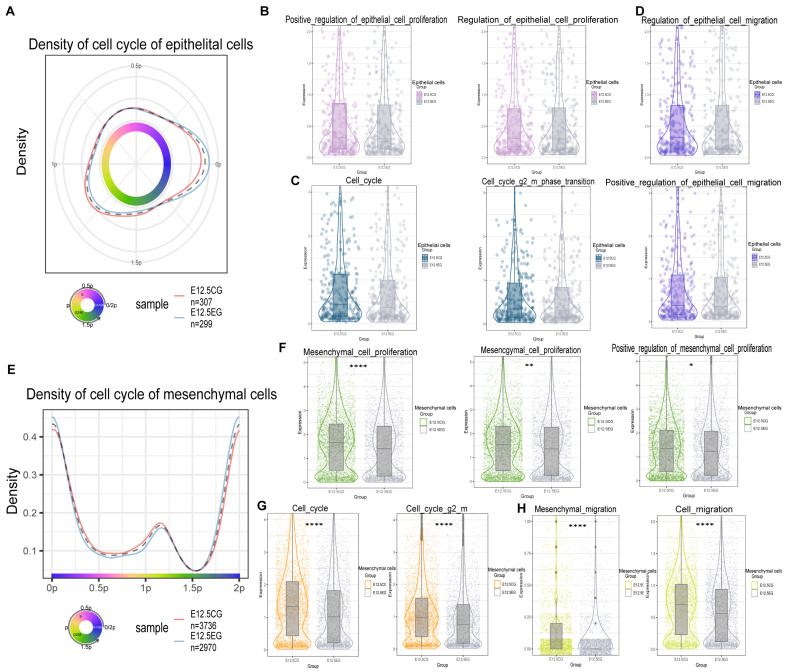
Cell cycle, proliferation, and migration assessments in mesenchymal and epithelial cells. (**A**) Comparison of scoring between control group and experimental group at different cell cycle stages in epithelial cells using “tricycle” package. (**B**) Epithelial cells proliferation-related pathway score in different groups. (**C**) Epithelial cells cell cycle-related pathway score in different groups. (**D**) Epithelial cells migration-related pathway score in different groups. (**E**) Comparison of scoring between control group and experimental group at different cell cycle stages in mesenchymal cells using “tricycle” package. (**F**) Mesenchymal cells proliferation-related pathway score in different groups. (**G**) Mesenchymal cells cell cycle-related pathway score in different groups. (**H**) Mesenchymal cells migration-related pathway score in different groups. Significance was indicated by “*”, “**” and “****” for *p* < 0.05, *p* < 0.01, and *p* < 0.0001, respectively.

The “tricycle” package was employed to evaluate cell cycle phases by analyzing gene expression profiles in both atRA-exposed and control groups. By scoring each cell based on the expression of G2/M, G2, S, G1/S, and M/G1 phase marker genes, cells were classified into the different phases or designated as undetermined. In Figure 3A,E, the most crucial phase of the cell cycle, G2/M, which represents the cell’s proliferative potential, is denoted by the yellow-green section. Notably, the atRA-exposed group exhibited significant inhibition in the G2/M phase in both cell types, indicating a suppression of cell proliferation (Figure 3A,E). Subsequently, the proliferation-related pathways in mesenchymal and epithelial cells were analyzed. The results revealed a slight inhibition of proliferation and cell cycle progression in epithelial cells within the atRA-exposed group, but without statistical significance (Figure 3B,C). At the same time, mesenchymal cells demonstrated a similar trend in pathway scores, with great significance (Figure 3F,G). Considering that previously identified DEGs were enriched in cell migration pathways in both cell types, the migration-related pathways were also evaluated. The findings indicated that epithelial cell migration exhibited a slight inhibition without significance (Figure 3D), while mesenchymal cells exhibited a highly significant inhibition of migration (Figure 3H).

### 3.4. All-Trans Retinoic Acid Intricately Regulates the Wnt and Bmp Pathways Between Mesenchymal and Epithelial Cells

**Figure 4:**
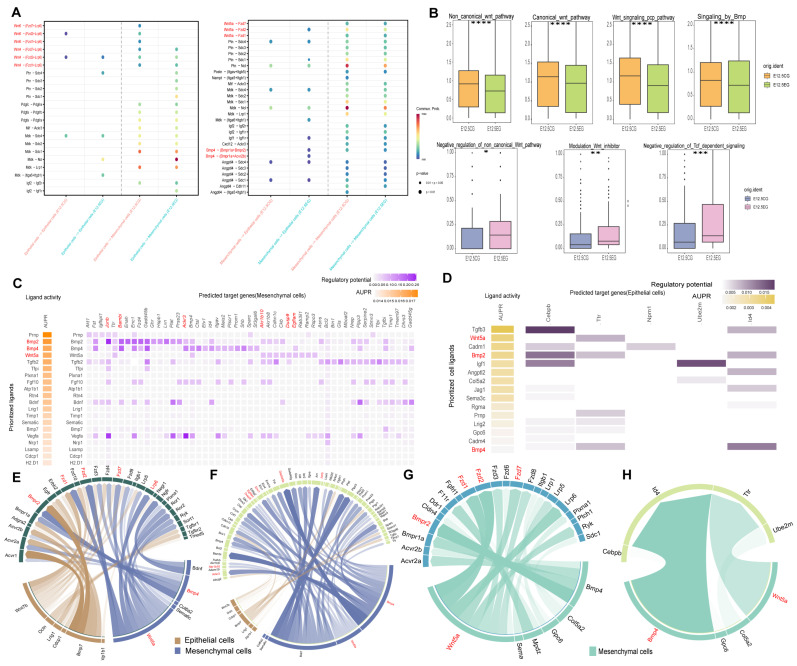
Cell interaction between mesenchymal and epithelial cells. (**A**) Sender design for epithelial cells, epithelial and mesenchymal cells as signal receptors (left); Sender design for mesenchymal cells, epithelial and mesenchymal cells as signal receptors (right). (**B**) Wnt and Bmp pathway scores in mesenchymal cells between different groups (orange and green); Wnt pathway score in epithelial cells between different groups (blue and pink). (**C**) Heatmap showing the cellular regulatory potential and ligand activity of mesenchymal cells to other cells. (**D**) Heatmap showing the cellular regulatory potential and ligand activity of epithelial cells to other cells. (**E**) Circos plots showing ligand–receptor pairs in mesenchymal cells with regulatory potential value. (**F**) Circos plots showing ligand–target gene pairs in mesenchymal cells with regulatory potential value. (**G**) Circos plots showing ligand–receptor pairs in epithelial cells with regulatory potential value. (**H**) Circos plots showing ligand–target gene pairs in epithelial cells with regulatory potential value. Significance was indicated by “*”, “**”, “***” and “****” for *p* < 0.05, *p* < 0.01, *p* < 0.001, and *p* < 0.0001, respectively.

To explore the dynamics of signaling pathways, the “CellChat” package was utilized to explore cell–cell communication between mesenchymal and epithelial cells. When mesenchymal cells acted as receptor cells, significant inhibition was observed in the Wnt5a, Wnt6, and Bmp4 pathways, along with a minor activation of the Wnt4 pathway. Conversely, when epithelial cells functioned as receptors, the Wnt4 and Wnt6 pathways displayed similar patterns to those noted in mesenchymal cells; however, the Bmp4 and Wnt5a pathways showed a slight activation (Figure 4A). In epithelial cells, both Wnt pathways were significantly activated in the experimental group, whereas the Bmp pathway showed no significant difference (Figure 4B and Figure S1D). Notably, atRA exerts complex regulatory effects on the Wnt and Bmp pathways in both cells. Additionally, “NicheNet” was employed to infer ligand–target regulatory potential. Analysis of receptor cells indicated high Wnt5a and Bmp2/4 ligand activity, irrespective of whether epithelial or mesenchymal cells were the primary receptors (Figure 4C,D,F,H). Subsequently, ligands with high signaling activity and receptors showing strong interactions were investigated across different cell types. The high-activity ligands Wnt5a and Bmp4 in both cells consistently interacted with a shared set of receptors with strong interactions, namely Fzd1, Fzd2, Fzd7, Lrp6, and Bmpr2 (Figure 4E,G). Thereafter, key target genes were identified, including *Junb*, *Bambi*, *Enc1*, *Gadd45b*, *Ackr3*, *Akr1b10*, *Dusp9*, and *Egflam* in mesenchymal cells (Figure 4C,F), and *Cebpb*, *Ttr*, *Npm1*, *Ube2m*, and *Id4* in epithelial cells (Figure 4D,H). These genes are not always associated with the cleft palate area, but they are linked to tumor migration and proliferation in humans and mice [21,22,23,24,25,26,27,28,29,30]. This study indicates the presence of an intricate crosstalk between the Wnt signaling pathways and the TGF-β superfamily Bmp signaling pathways in mesenchymal and epithelial cells.

### 3.5. hdWGCNA Analysis Revealed Key Gene Modules and Core Transcription Factors Related to the All-Trans Retinoic Acid-Exposed Group

**Figure 5:**
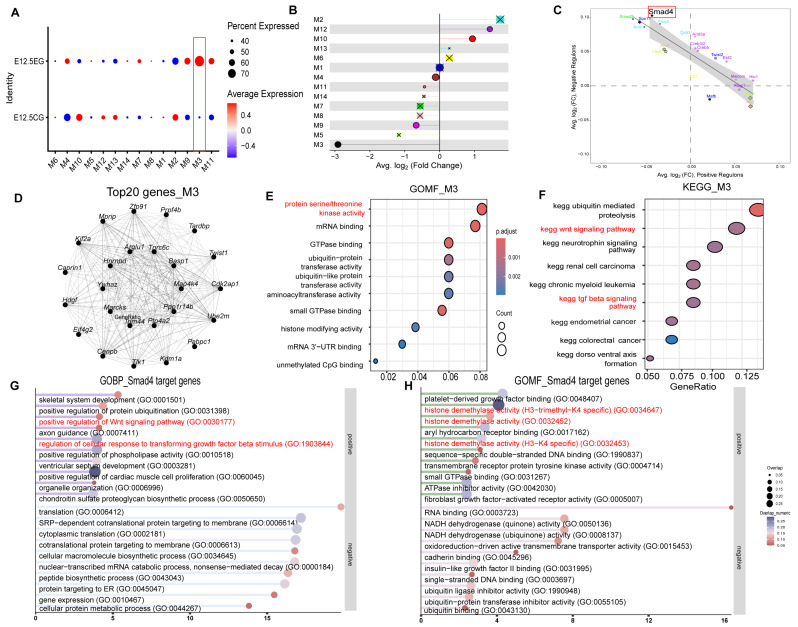
hdWGCNA analysis in mesenchymal cells. (**A**) Fourteen differently expressed gene modules in two groups. (**B**) Differential module eigengene (DME) analysis revealing module 3 is highly correlated with atRA-exposed group (The circles in different colors represent distinct gene modules, while the ‘X’ marks indicate that there is no statistically significant difference in the gene module between the two groups). (**C**) Differential regulon analysis revealing Smad4 is significantly differentially expressed TF in module 3 (Diamonds represent TFs that are also significantly differentially expressed, while circles are not differentially expressed;genes highlighted in different colors represent differentially expressed transcription factors within distinct gene modules). (**D**) Co-expression network plot showing top 20 hub genes in module 3. (**E**) GOMF enrichment analysis of module 3 genes. (**F**) KEGG enrichment analysis of module 3 genes. (**G**) GOBP enrichment analysis of positive and negative target genes of Smad4. (**H**) GOMF enrichment analysis of positive and negative target genes of Smad4.

**Figure 6:**
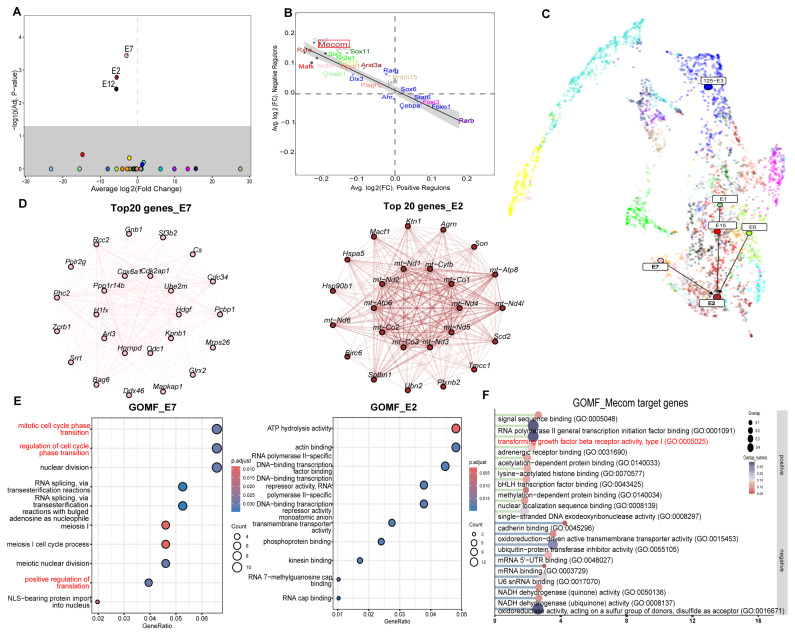
hdWGCNA analysis in epithelial cells. (**A**) Differential module eigengene (DME) analysis revealed that epithelial cell modules 2, 7, and 12 are highly correlated with atRA-exposed group (The circles in different colors represent distinct gene modules). (**B**) Differential regulon analysis revealing Mecom is significantly differentially expressed TF in epithelial cell module 2. (**C**) Module Regulatory Network Plot analysis revealing epithelial cell module 2 is positively regulated by epithelial cell module 7. (**D**) Co-expression network plot showing top 20 hub genes in epithelial cell module 7 (left) and epithelial cell module 2 (right). (**E**) GOBP enrichment analysis of epithelial cell module 7 (left) and GOMF enrichment analysis of epithelial cell module 2 (right) genes. (**F**) GOMF enrichment analysis of positive and negative target genes of Mecom.

Considering the primary effects of atRA on mesenchymal and epithelial cells, the “hdWGCNA” package was used to identify the modules most significantly correlated within the atRA-exposed group. The analysis pipeline is detailed in the Supplementary Materials (Figure S2A,B). Our analysis revealed 14 gene modules in mesenchymal cells (Figure S2C), demonstrating differential expression across the two groups (Figure 5A,B). Notably, module 3 (M3) exhibited a strong positive correlation with atRA exposure (Figure 5A,B). Subsequently, genes within M3 were subjected to functional enrichment analyses using KEGG and GOMF (Figure 5D–F). The results indicated enrichment in the Wnt signaling pathway, TGF-β signaling pathway, and GTPase binding, among others. Differential regulons analysis identified Smad4 as the principal differentially expressed transcription factor in the M3 (Figure 5C). Subsequently, GOMF and KEGG pathway enrichment analyses were conducted on the target genes of Smad4. These analyses revealed significant involvement in the Wnt and TGF-β pathways, as well as methylation-related pathways (Figure 5G,H). Meanwhile, the same analytical framework was applied to epithelial cells (Figure S3A–D). Our results indicated that Modules 2, 7, and 12 (E2,E7,E12) exhibited significant differences between the two groups (Figure 6A). Notably, E2 demonstrated a marked difference between the groups, with the transcription factor Mecom identified as significantly differentially expressed (Figure 6A,B). Subsequent GOMF analysis of E2 genes revealed involvement in ATP-related processes and transcriptional inhibition (Figure 6E). Furthermore, GOMF analysis of Mecom’s target genes indicated enrichment in TGF-β-related pathways (Figure 6F). In addition, the Module Regulatory Network Plot analysis was conducted to identify the relationships among these three modules. Our findings indicated that E7 exerts a positive regulatory effect on E2, with a significant difference observed between the two groups (Figure 6A,C). Subsequently, the gene components of E7 were examined, which exhibited a high degree of similarity to those in M3 within mesenchymal cells (Figure 5D and Figure 6D). Further GOMF and GOBP analyses were conducted on E7, revealing significant involvement in cell cycle processes and GTPase-related processes (Figure 6E and Figure S3E). Notably, the key transcription factor and key modules identified in both cell types focused on the Wnt and TGF-β pathways.

### 3.6. Ppp1r114b Is an Important Gene Mediating All-Trans Retinoic Acid’s Effect on Palatal Shelves Development

**Figure 7:**
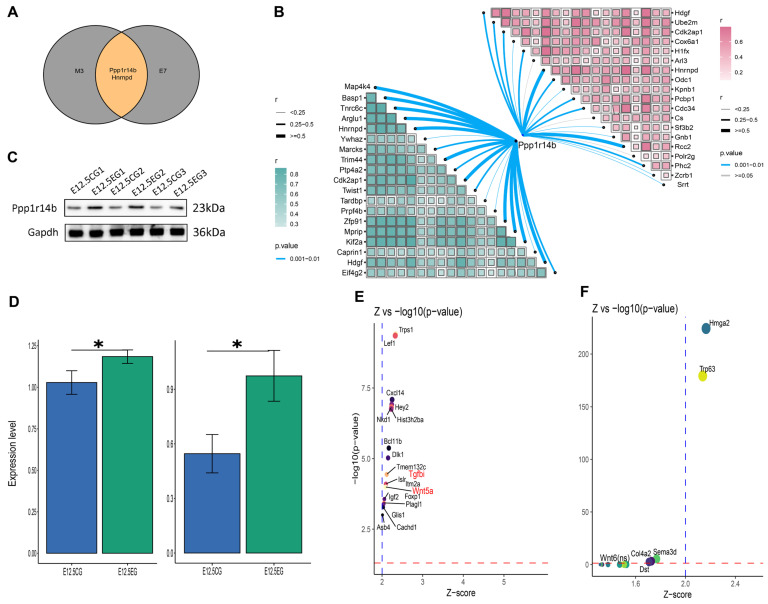
Ppp1r14b’s expression and function. (**A**) Venn diagram of the top 10 genes in M3 and top 10 genes in E7. The overlap, indicated in orange, signifies the genes common to both modules. (**B**) Gene correlation analysis between *Ppp1r14b* and top 20 genes in M3 and top 20 genes in E7. (**C**) Western blot of Ppp1r14b and Gapdh in control and atRA-exposed groups. (**D**) Statistical analysis of qRT-PCR (left) and Western blot (right). (**E**) Simulation of *Ppp1r14b* knock out in mesenchymal cells (Genes are considered significant only when the Z-score exceeds 2 and the *p*-value is less than 0.05). (**F**) Simulation of *Ppp1r14b* knock out in epithelial cells. Significance was indicated by “*”for *p* < 0.05.

The top 10 hub genes between M3 and E7 were intersected. *Ppp1r14b* and *Hnrnpd* emerged as intersecting genes (Figure 7A). Subsequently, the “scTenifoldKnk” package was utilized to develop virtual gene knockout models for these genes in both mesenchymal and epithelial cells. Our findings indicated that only the *Ppp1r14b* gene enabled the creation of meaningful perturbation models. Notably, the top 20 genes most affected by *Ppp1r14b* knockout included *Wnt5a*, *TGFbi*, *Wnt6*, and others, in both cell types (Figure 7E,F). Correlation analysis between *Ppp1r14b* and the top 20 hub genes of M3 and E7 demonstrated a positive correlation with both gene sets (Figure 7B). Based on these observations, *Ppp1r14b* may influence the effect of atRA on palatal shelves development. Therefore, qRT-PCR and Western blot analysis of Ppp1r14b were conducted in both the control and atRA-exposed groups. The results revealed significant overexpression of Ppp1r14b in the atRA-exposed group, which is consistent with the results of the bioinformatics analysis (Figure 7C,D).

## 4. Discussion

Excessive consumption of atRA may result in cleft palate due to its teratogenic effects [31]. Previous research on cleft palate models induced by atRA primarily focused on later stages of palatal shelves development, overlooking the initial development phase at E12.5. This study employed single-cell sequencing to explore the effects of atRA on palatal shelves at E12.5, identifying potential biomarkers and intervention targets for early detection of cleft palate.

Our research shows that atRA exposure inhibits mesenchymal cell proliferation at E12.5, which is consistent with previous studies [32,33,34]. Moreover, mesenchymal cell migration was significantly suppressed. In epithelial cells, atRA also demonstrated an inhibitory effect on their proliferation at E12.5, while exerting a negligible effect on migration. Although this inhibition was not statistically significant, it is possible that the limited number of epithelial cells in both the experimental and control groups contributed to this outcome. Thereafter, the underlying pathway mechanisms were investigated.

Our findings indicated that the Wnt and TGF-β pathways are the principal pathways influenced by atRA. The Wnt pathway can be categorized into the canonical β-catenin-dependent pathway and the non-canonical pathways, the latter involving multiple serine/threonine protein kinase components [35]. Wnt5a interacts with Fzd7 and may mediate the planar cell polarity (PCP) pathway and subsequently activate c-Jun N-terminal kinase (JNK), which is a branch of the non-canonical Wnt signaling pathways [36]. Previous studies have suggested that inhibition of the Wnt5a pathway leads to the inhibition of mesenchymal cell migration or disorder via the JNK pathway [37]. In this study, inhibition of the Wnt5a pathway is also a fundamental mechanism by which atRA impedes the migration and proliferation of mesenchymal cells in the secondary palate at E12.5.

Furthermore, research indicates that the Wnt6-Lrp6 pathway is inhibited, while the Wnt4-Lrp6 pathway is activated in mesenchymal cells. The inhibition of the canonical Wnt pathway mediated by Wnt6 may significantly suppress mesenchymal cell proliferation [38]. However, research regarding the influence of the Wnt4 pathway on palatal mesenchymal cells remains scarce. Our study indicates that the Wnt4 pathway is activated at E12.5 in mesenchymal cells. Obviously, atRA concurrently modulates both canonical and non-canonical Wnt pathways, thereby impacting mesenchymal cells.

In addition, this study proposes that atRA inhibits epithelial cell proliferation. atRA may induce alterations in the pathway, primarily affecting Wnt5a, with Wnt4 being activated and Wnt6 significantly inhibited. Hence, the inhibitory effect of atRA on epithelial cells may be mediated by both canonical and non-canonical Wnt signaling pathways.

Previous research has established that Bmp signaling genes play a crucial role in the development of palatal shelves, particularly by regulating mesenchymal cell proliferation [39,40]. In this study, the TGF-β signaling pathway demonstrated substantial changes in the atRA-exposed group. Notably, Bmp2/4, a member of the TGF-β superfamily, was among the most prominent factors. The present study suggests that Bmp4-Bmp1ra was significantly inhibited in mesenchymal cells of the atRA-exposed group, and that both Bmp2 and Bmp4 are highly active ligands (Figure 4C,D,F,H). Bmp2 and Bmp4 exhibit pronounced synergistic effects on their functional roles and expression regions. Both Bmp2 and Bmp4 independently stimulated cell proliferation in palatal mesenchymal cells in vitro [41,42]. Inhibition of mesenchymal cell proliferation is hypothesized to be linked to the suppression of the Bmp2/4- BmprIa signaling pathway. Previous studies show that following the inhibition of Wnt5a, a significant downregulation of Bmp2/4 was observed in the palate [43,44]. These findings indicate a synergistic interaction between Wnt5a and Bmp2/4, which collectively regulate the proliferation of mesenchymal cells.

Smad4 is a key transcription factor in the Bmp signaling pathway [45]. The current study indicates that Samd4 functions as a pivotal transcription factor in mesenchymal cells between the two groups. Similarly, a previous study reported that atRA leads to the downregulation of the Smad signaling pathway and the inhibition of mesenchymal cell proliferation [46,47]. In contrast to mesenchymal cells, epithelial cells activated the Bmp2/4 pathway in the atRA-exposed group. Our hdWGCNA analysis indicated that the transcription factor Mecom was significantly changed in epithelial cells. The target genes of Mecom were found to be enriched in the TGF-β pathway; notably, the Bmp/Smad pathway also plays a crucial regulatory role in epithelial cells. Previous research has reported overexpression of Bmp2 signals through the BmprIa receptor and Smad-dependent pathway, resulting in the disruption of epithelial cell integrity in the palate [48]. Due to Bmp2’s synergistic effects with Bmp4, we hypothesized that Bmp4 signals’ activation may also influence the survival and growth of epithelial cells.

In addition, we found *Ppp1r14b* as a hub gene shared between mesenchymal and epithelial cell key gene modules related to atRA exposure, with its differential expression showing highly significant results (Figure 1F, Figure 5D and Figure 6D). These findings suggest that Ppp1r14b may play a pivotal role in mediating the effects of atRA during early palatal shelves development. PCR and Western blot validation further confirmed significant differential expression between the control and experimental groups (Figure 7C,D). Additionally, virtual knockout experiments using the scTenifoldKnk algorithm revealed that the knockdown of *Ppp1r14b* notably affected the expression of *Tgfbi*, *Wnt5a*, and *Wnt6* (Figure 7E,F), suggesting that Ppp1r14b may influence both Wnt and TGF-β signaling pathways. Notably, the association between this gene and palate development has not been previously documented. Ppp1r14b is a member of the PP1 family [49,50], which is involved in various biological processes, including glycogen metabolism, cell cycle progression, protein synthesis, and apoptosis [51]. Upregulation of Ppp1r14b inhibits PP1 expression [52], enhancing proliferation and invasion across various tumor cells [53,54,55]. Our study indicates that Ppp1r14b is highly expressed in the atRA-exposed group, a group that inhibits the proliferation of mesenchymal and epithelial cells, a finding that contradicts previous research on tumor biology. The specific function of Ppp1r14b on the mesenchymal and epithelial cells during palatal shelves development requires further investigation.

Although this study provides valuable insights into the effects of atRA on secondary palatal shelves development at E12.5 through single-cell sequencing and experimental investigation, several limitations must be acknowledged. Firstly, the study exclusively explores the impact of atRA on cleft palate formation in mice, limiting the generalizability of the findings to humans. Secondly, the sample size of sequencing data is relatively small; larger-scale sequencing with more samples would yield more robust and meaningful results. Finally, while the study suggests that Ppp1r14b may influence Wnt and TGF-β signaling pathways, in vivo and in vitro experiments to validate these findings were not conducted, which will be an important direction for future research.

## 5. Conclusions

atRA is a well-established teratogen associated with the induction of cleft palate in humans and mice. This study elucidates the impact of atRA on mesenchymal and epithelial cells during the initial phases of palatal shelves development. The results indicate that atRA influences cell proliferation and migration, mainly through the Wnt and TGF-β pathways. In addition, *Ppp1r14b* may have an important gene interaction with these pathways during atRA exposure, inducing cleft palate. All in all, this study offers novel insights for future research on cleft palate pathogenesis.

## Data Availability

The data presented in this study are openly available in the Sequence Read Archive (SRA), reference numbers PRJNA989673 and PRJNA1309318.

## Supplementary Materials

The following supporting information can be downloaded at: https://www.mdpi.com/article/10.3390/biomedicines13112836/s1, Table S1: Primer of Ppp1r14b and Dapdh; Figure S1: Functional pathway enrichment analysis in mesenchymal cells and epithelial cells and Bmp pathway score in epithelial cells; Figure S2: hdWGCNA pipeline in mesenchymal cells; Figure S3: hdWGCNA pipeline in epithelial cells.

## Abbreviations

The following abbreviations are used in this manuscript:

atRA	All-trans retinoic acid
E12.5	embryonic day 12.5
E12.5–E16.5	embryonic days 12.5–16.5
UMI	unique molecular identifier
DEGs	Differentially expressed genes
GOBP	Gene Ontology biological process
GOMF	Gene Ontology molecular function
KEGG	Kyoto Encyclopedia of Genes and Genomes
qRT-PCR	Quantitative Real-Time Polymerase Chain Reaction
SDS-PAGE	Sodium dodecyl sulfate polyacrylamide gel electrophoresis
PVDF	Polyvinylidene Fluoride
PCP	planar cell polarity
JNK	c-Jun N-terminal kinase
