# Supplementary material for: All-Trans Retinoic Acid Impacts Early Palatal Shelves Development via the Wnt and TGF-β Signaling Pathways
Source: Biomedicines. 2025 Nov 20;13(11):2836. doi: 10.3390/biomedicines13112836 (PMC12650183; doi:10.3390/biomedicines13112836)
Supplement: Supplementary file 1 [file biomedicines-13-02836-s001.zip › biomedicines-3889304-supplementary.pdf]

Table S1. Primer of Ppp1r14b and Gapdh

Gene	Sequence	
	Forward	Reward
Ppp1r14b	AAGACAGAGAAAGCGGCAGCGGC	AATCGACGACCTTTCCGACATAG
Gapdh	GAGCCAAAAGGGTCATCATCT	AGGGGCCATCCACAGTCTTC

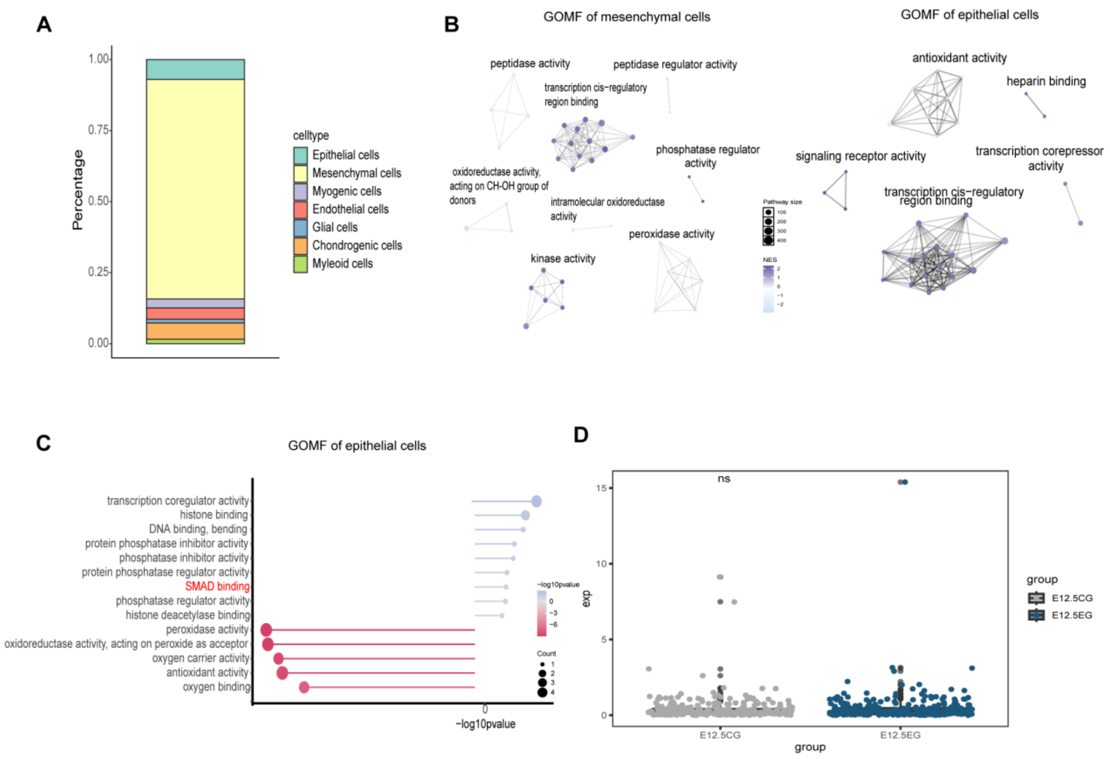

Figure S1. Functional pathway enrichment analysis in mesenchymal cells and epithelial cells and Bmp pathway score in epithelial cells

(A) Cell proportion diagram.

(B) GOMF enrichment analysis of mesenchymal cells(left) and epithelial cells(right).

(C) GOMF enrichment analysis of epithelial cells.

(D) Bmp pathway score in epithelial cells.

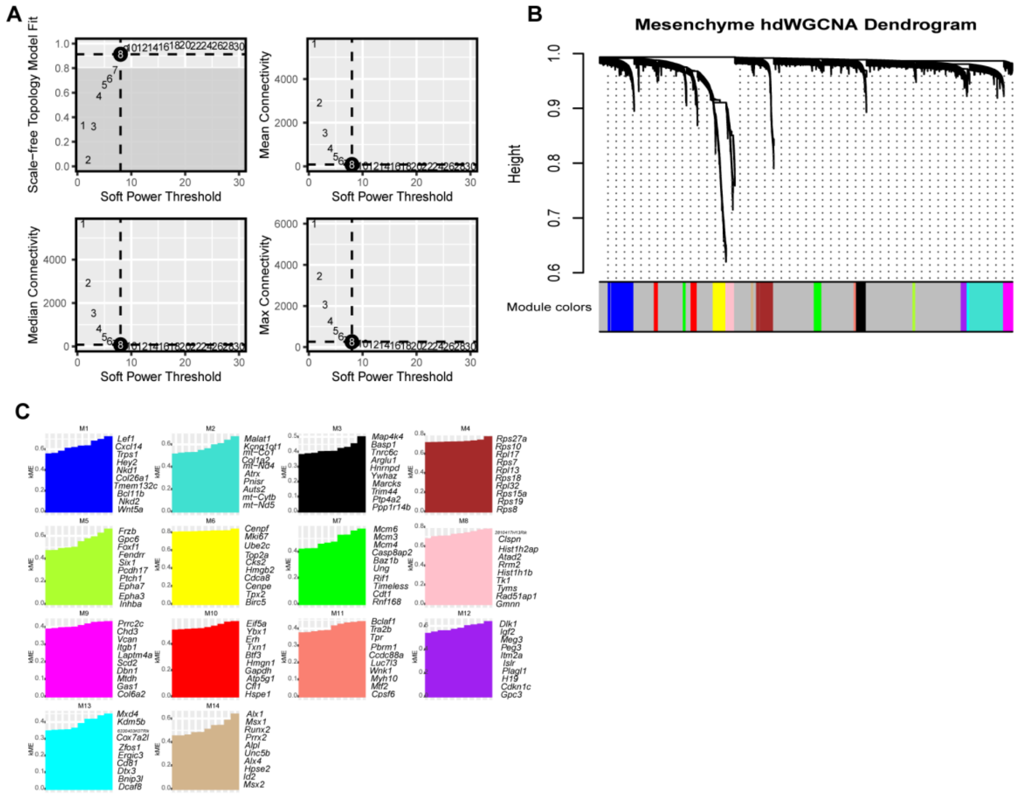

Figure S2. hdWGCNA pipeline in mesenchymal cells.

(A)Optimal soft threshold selection in mesenchymal cells.

(B)Dendrogram showing hdWGCNA analysis of the mesenchymal cells.

(C)Ridge plot showing the 14 gene modules identified by co-expression analysis.

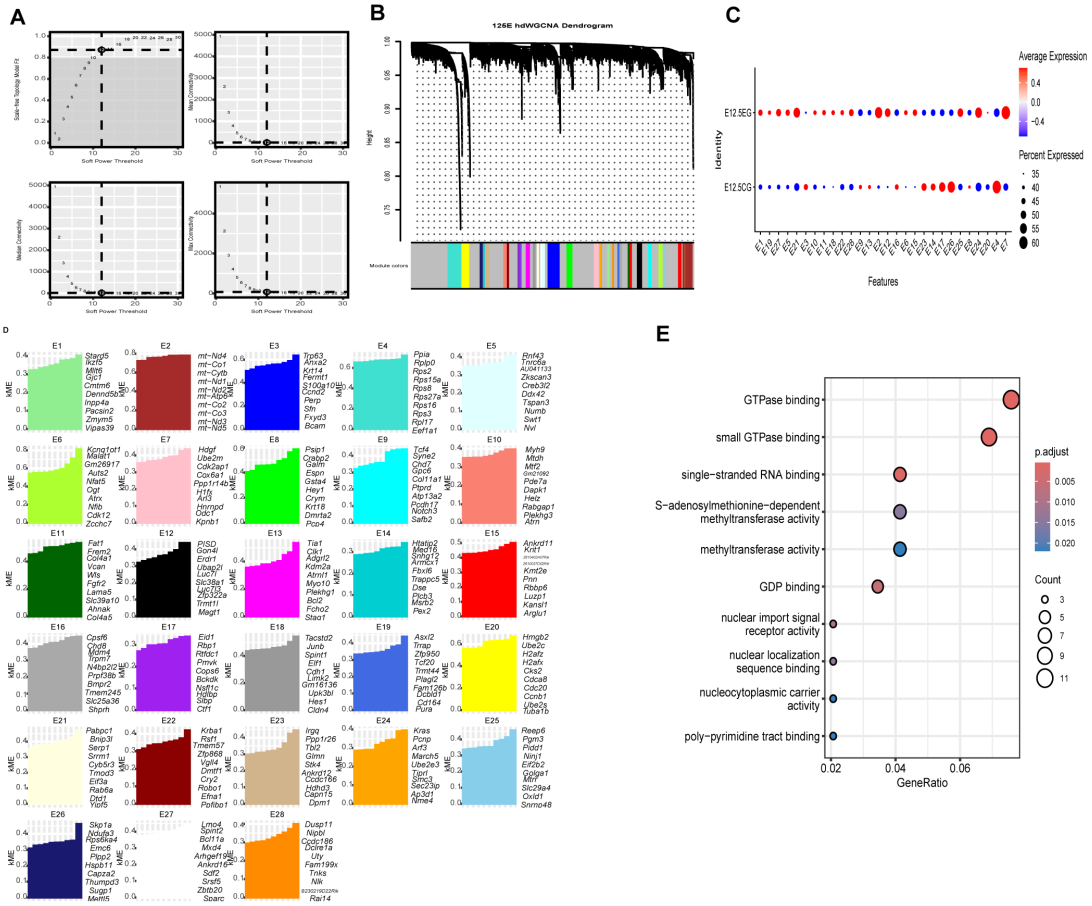

Figure S3: hdWGCNA pipeline in epithelial cells.

(A)Optimal soft threshold selection in epithelial cells.

(B)Dendrogram showing hdWGCNA analysis of the epithelial cells.

(C)Ridge plot showing the 28 gene modules identified by co-expression analysis.

(D)28 gene modules different expressed in different groups.

(E)GOMF enrichment analysis of module 7 genes.
